# Curcumol β-cyclodextrin inclusion complex enhances radiosensitivity of esophageal cancer under hypoxic and normoxic condition

**DOI:** 10.1007/s11604-023-01446-7

**Published:** 2023-05-25

**Authors:** Meng Su, Xiaolin Ren, Dexi Du, Huijuan He, Dahai Zhang, Raoying Xie, Xia Deng, Changlin Zou, Haizhou Zou

**Affiliations:** 1 Department of Radiation Oncology, The First Affiliated Hospital of Wenzhou Medical University, Nanbaixiang Street, Wenzhou, 325000 Zhejiang People’s Republic of China; 2 Department of Radiation Oncology, Lishui Central Hospital, Lishui, Zhejiang People’s Republic of China; 3 Department of Radiation Oncology, Quzhou People’s Hospital, Quzhou, Zhejiang People’s Republic of China; 4 Department of Radiation Oncology, Dongyang People’s Hospital, Jinhua, Zhejiang People’s Republic of China; 5 Derpartment of Medical Oncology, Wenzhou Hospital of Chinese Medicine, No. 9 Jiaowei Street, Wenzhou, 325000 Zhejiang People’s Republic of China

**Keywords:** Curcumol, Esophageal cancer, Radiosensitivity, Apoptosis, Cell cycle

## Abstract

**Purpose:**

Radiotherapy is an indispensable treatment for esophageal cancer (EC), but radioresistance is not uncommon. Curcumol, as an active extract from traditional Chinese medicines, has been reported to have antitumor activity in various types of human tumor cells. However, its reversal of radioresistance has been rarely reported.

**Materials and methods:**

In the present study, curcumol was prepared as an inclusion complex with β-cyclodextrin. EC cell lines were treated with radiation and curcumol β-cyclodextrin inclusion complex (CβC), and the effect of radiosensitization of CβC was investigated in vitro and in vivo. The in vitro experiments included cell proliferation assay, clonogenic survival assay, apoptosis assay, cell cycle assay, and western blot assay.

**Results:**

The in vitro data revealed that CβC and irradiation synergistically inhibited the proliferation, reduced the colony formation, promoted the apoptosis, increased the G2/M phase, inhibited DNA damage repair, and reversed the hypoxia-mediated radioresistance of EC cells to a greater extent than did CβC alone or irradiation alone. The sensitization enhancement ratios (SERs) were 1.39 for TE-1 and 1.48 for ECA109 under hypoxia. The SERs were 1.25 for TE-1 and 1.32 for ECA109 under normoxia. The in vivo data demonstrated that the combination of CβC and irradiation could inhibit tumor growth to the greatest extent compared with either monotherapy alone. The enhancement factor was 2.45.

**Conclusion:**

This study demonstrated that CβC could enhance radiosensitivity of EC cells under hypoxic and normoxic condition. Thus, CβC can be used as an effective radiosensitizer for EC.

## Introduction

Esophageal cancer (EC) is one of the most common malignant tumors, with high morbidity and mortality. According to the latest global cancer statistics, the number of new cases of EC was 473000, ranking 10th; death toll of EC was 436000, ranking 7th [1]. In 2020, EC was estimated to account for more than 600,000 new cancer cases and 540,000 deaths in the world [2]. EC has two main histological subtypes: esophageal squamous cell carcinoma (ESCC) and esophageal adenocarcinoma (EA). The incidence of EC has obvious regional differences, and the incidence in East Asia is much higher than that in other regions. ESCC is more common in high incidence regions, such as China [1, 2]. Radiation therapy is the primary treatment for ESCC since this entity is highly radiosensitive, however radioresistance is still not uncommon.

The presence of radioresistant cells significantly reduces the efficacy of radiotherapy. Even if the radiation dose for EC is increased, the local control and overall survival are not improved, but radiotherapy-related side effects are increased [3]. Therefore, clearing radioresistant cells or reversing radiotherapy resistance has become the key to improve the efficacy of radiotherapy for EC. Radiosensitivity is affected by multiple factors, including hypoxia, apoptosis, cell cycle, and DNA damage repair. Modulation of only one factor cannot effectively sensitize radiotherapy. Thus, it is necessary to find an effective and safe radiosensitizer to modulate multiple radioresistance factors.

The genus *Curcuma* is in the family *Zingiberaceae.* Curcuma, as a traditional medicine, has been widely used for anti-cancer, anti-hepatic fibrosis, anti-fungal, anti-viral, and anti-inflammatory [4, 5]. Curcumol, as an important active component of Curcuma, extracts from numerous plants of family *Zingiberaceae*, has the effects of inhibiting tumor growth, cell cycle arrest, and promoting apoptosis in a variety of tumor cells via targeting the mitogen-activated protein kinase (MAPK)/extracellular signal-regulated kinase (ERK), phosphatidylinositol 3-kinase (PI3K)/protein kinase B (Akt), and nuclear factor kappa-light-chain-enhancer of activated B cells (NF-κB) signaling pathways [4, 5]. β-cyclodextrin, as a stabilizing agent, greatly increases the stability and solubility of curcumol [6, 7]. And curcumol β-cyclodextrin inclusion complex (CβC) has been proved to retain obvious antitumor activity [8].

Nevertheless, doubts still existed about whether CβC can enhance radiosensitivity of EC under hypoxic and normoxic condition. Based on this consideration, the aim of this study was to identify whether CβC is a potential radiosensitizer for EC cells.

## Materials and methods

### Reagents and antibodies

Curcumol was purchased from the National Institute of Control of Pharmaceutical and Biological Products (Beijing, China; CAS: 458-37-7). β-cyclodextrin was purchased from the Tianjin Guangfu Fine Chemical Research Institute (Tianjin, China; CAS: 68168-23-0). Curcumol was prepared as an inclusion complex with β-cyclodextrin by saturated solution method, and the inclusion complex was verified by infrared spectroscopy according to previous study [8]. CβC was dissolved in dimethyl sulfoxide (DMSO, Sigma, St. Louis, USA). RPMI-1640 medium, fetal bovine serum (FBS, Australia), and penicillin/streptomycin were purchased from Gibco (Thermo Fisher Scientific, Inc., Waltham, MA, USA). The Cell Counting Kit‑8 (CCK‑8) was purchased from Dojindo Laboratories (Tokyo, Japan). The Annexin V‑fluorescein isothiocyanate (FITC)/propidium iodide (PI) apoptosis detection kit and PI/RNase staining buffer were purchased from Becton–Dickinson (BD) Biosciences (San Jose, CA, USA). The bicinchoninic acid (BCA) protein assay kits, and enhanced chemiluminescence (ECL) kits were purchased from Beyotime Biotechnology (Shanghai, China).

Primary antibodies against β-actin (#4970), Bcl-2 (B cell lymphoma‑2, #4223), Bax (Bcl‑2‑associated X, #14796), caspase-3 (#9662), cleaved caspase-3 (#9664), HIF-1α (hypoxia-inducible factor 1α, #36169), PI3K (#4292), phospho-PI3K (p-PI3K, #4228), Akt (#4691), phospho-Akt (p-Akt, #4060), mTOR (mammalian target of rapamycin, #2983), and phospho-mTOR (p-mTOR, #5536) were purchased from Cell Signaling Technology (Beverly, MA, USA). Antibodies against cyclin B1 (sc‑7393), CDK1 (cyclin-dependent kinase 1, sc‑53219), Ku86 (sc‑5280), Ku70 (sc‑17789), Rad51 (sc‑133089), and Rad54 (sc-374598) were purchased from Santa Cruz Biotechnology (Santa Cruz, CA, USA). Antibody against VEGF (vascular endothelial growth factor, ab46154) was purchased from Abcam (Cambridge, MA, USA). Horseradish peroxidase (HRP)-conjugated goat anti-rabbit IgG H&L secondary antibody (ab205718) was purchased from Abcam (Cambridge, MA, USA).

### Cell culture and irradiation treatment

Two human esophageal cancer cell lines, TE-1 and ECA109, were purchased from the Cell Bank of Type Culture Collection of Chinese Academy of Sciences (Shanghai, China) and cultured in RPMI-1640 with 10% FBS and 1% penicillin/streptomycin. The cells were incubated in a humidified atmosphere at 37 °C with 5% CO_2_ (normoxic condition) or a complex air of 1% O_2_, 94% N_2_, and 5% CO_2_ (hypoxic condition). Exponentially growing cells were used for all the experiments.

Irradiation was delivered as a single dose ranging from 0 to 8 Gy at a dose rate of 200 cGy/min using a 6-MV X-ray linear accelerator (Elekta AB, Stockholm, Sweden) at room temperature. The source to cell distance was 100 cm, and the field size was 20 × 20 cm.

### Cell proliferation assay

Cells were seeded into 96-well flat plates at a density of 6000 cells/well, and treated with various concentrations of CβC ranging from 0 to 640 μg/ml for 24, 48, and 72 h. Each group contained 6 parallel wells. Following the indicated time of treatment, 10 μl of CCK‑8 solution was added to each well. After incubating for another 4 h at 37 °C, the absorbance was measured at 450 nm.

Cells were seeded into 96-well flat plates at a density of 6000 cells/well. The cells were irradiated at 0, or 8 Gy after CβC treatment (0, 10 or 80 μg/ml) for 24 h under hypoxic and normoxic condition. Then, the cells were cultured for another 48 h under previous condition. After that, 10 μl of CCK‑8 solution was added to each well. After incubating for another 4 h at 37 °C, the absorbance was measured at 450 nm.

### Clonogenic survival assay

Cells were seeded into 6-well flat plates at different densities ranging from 300 to 4000 cells/well, and treated with CβC (0, 10 or 80 μg/ml) for 24 h under hypoxic and normoxic condition. Then, the cells were irradiated at 0, 2, 4, 6, or 8 Gy and cultured for another 12 days. After that, the cells were fixed with methanol and stained with crystal violet for 0.5 h. The colonies containing more than 50 cells were counted under microscopy. The survival curves were fitted according to the single-hit multi-target model (survival fraction (SF) = 1 ‑ (1 ‑ exp(‑*D*/*D*_0_))^*n*^) by using GraphPad Prism 8.0 (GraphPad Software Inc., San Diego, CA, USA). D_0_ represented the mean lethal dose; D_q_ represented the quasi-domain dose; SF_2_ represented the survival fraction at 2 Gy. The sensitization enhancement ratio (SER) was calculated as the ratio of D_0_ control group value divided by D_0_ experimental group value.

### Apoptosis assay

Cells were seeded into 6-well flat plates at a specific density. The cells were irradiated at 0, or 8 Gy after CβC treatment (0, 10 or 80 μg/ml) for 24 h under hypoxic and normoxic condition. Then, the cells were cultured for another 48 h under previous condition. Subsequently, all cells of each group were collected by trypsin solution, washed twice with cold PBS, suspended in binding buffer, and labeled with Annexin V‑FITC and PI according to the protocol of manufacturer. Finally, all samples were analyzed by flow cytometer (Cytoflex, Beckman Coulter, USA).

### Cell cycle assay

Cells were seeded into 6-well flat plates at a specific density. The cells were irradiated at 0, or 8 Gy after CβC treatment (0, 10 or 80 μg/ml) for 24 h. Then, the cells were cultured for another 48 h. Subsequently, all cells of each group were collected by trypsin solution, washed twice with cold PBS, fixed in 70% ice‑cold ethanol overnight at 4 °C, and stained with PI/RNase according to the protocol of manufacturer. Finally, all samples were analyzed by flow cytometer (Cytoflex, Beckman Coulter, USA).

### Western blot assay

Cells were treated with CβC (0, 10 or 80 μg/ml), or CβC (0, 10 or 80 μg/ml) combined with irradiation (8 Gy) under hypoxic or normoxic condition. The cells were lysed by radio-immunoprecipitation assay (RIPA) buffer (Beyotime Biotechnology, Shanghai, China) with protease and phosphatase inhibitors (Roche, Indianapolis, IN, USA). The protein concentrations were measured by BCA protein assay kit. The protein samples were separated by SDS–polyacrylamide gel electrophoresis, and then transferred to polyvinylidene difluoride (PVDF) membranes (Millipore, Billerica, MA, USA). After blocking with Quickblock blocking buffer (Beyotime Biotechnology, Shanghai, China) for 0.5 h, the membranes were incubated with primary antibodies overnight at 4 °C and incubated with the corresponding HRP-conjugated secondary antibody for 2 h at room temperature. After that, the immunoblotted proteins were detected by ECL regents and visualized by ChemiDoc XRS imaging system (Bio‑Rad, Hercules, CA, USA). Finally, the relative levels of the proteins were quantified with ImageJ software (NIH, Bethesda, MD, USA).

### In vivo* experiment*

Male BALB/c nude mice (5-week-old), supplied by Beijing Weitong Lihua Laboratory Animal Technology Co., Ltd, were used in this study. ECA109 cells were resuspended in a 1:1 volume of PBS and Matrigel (BD Biosciences, San Jose, CA, USA), and then a suspension of 5 × 10^6^ cells was subcutaneously injected into the right forelimb armpit of each nude mouse. When the average tumor mass volume reached 100 mm^3^, the mice were randomly divided into 4 groups (n = 6): control group, CβC group, irradiation group, and combined treatment group. The nude mice in the control group and irradiation group were intraperitoneally injected with PBS every other day for a total of 2 weeks. The nude mice in the CβC group and combined treatment group were intraperitoneally injected with CβC (100 mg/kg) every other day for a total of 2 weeks. The nude mice in the irradiation group and combined treatment group were irradiated at a dose of 8 Gy on 8^th^ day after the start of PBS or CβC administration. Tumor volume (length diameter (mm) × width diameter (mm) ^2^ × 0.5) and body weight were measured every other day. On 28^th^ day after the start of PBS or CβC administration, the mice were sacrificed.

The tumor doubling time (DT, days) was calculated as: DT = *d* × lg2/lg(*V*_*d*_/*V*_*0*_), where d represented the length of time (days) between two measurements, V_d_ was the tumor volume at the time after treatment, and V_0_ was the tumor volume at the start of treatment. Absolute growth delay (AGD, days) was calculated as the DT of the treatment group minus the DT of the control group. Normalized growth delay (NGD, days) was calculated as the AGD of the combined treatment group minus the AGD of the CβC group. Enhancement factor (EF) was calculated as the NGD of the combined treatment group divided by the AGD of the irradiation group.

### Statistical analysis

All experiments were independently repeated three times. Data management was performed with SPSS 22.0 (IBM Corp., Armonk, NY, USA) and GraphPad Prism, Version 8.0 (GraphPad Software Inc., San Diego, CA, USA). All data were presented as mean ± standard deviation. All experimental data were tested for homogeneity of variance and normality. When the data conformed to homogeneous variance and normal distribution, t-test was used for comparison between two groups, one way ANOVA or two-way ANOVA was used for comparison among three or more groups. Non-parametric test was used for non-homogeneous variance or skewed distribution. Pairwise comparison in multiple groups was conducted using Bonferroni method for homogeneous variance and Dunnett's T3 method for non-homogeneous variance. Differences with p < 0.05 were considered to indicate statistical significance and all statistical tests were two-sided.

## Results

### CβC inhibits proliferation of EC cells

**Fig. 1:**
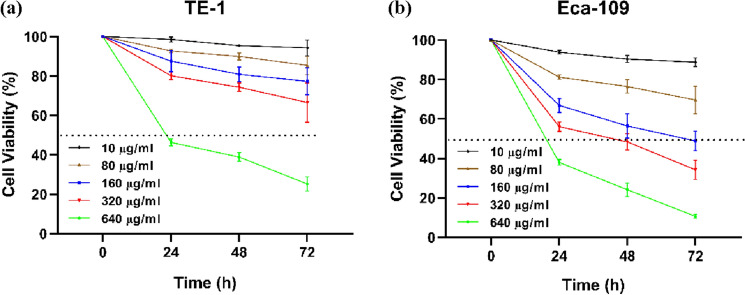
CβC inhibited the proliferation of EC cell lines including TE-1 (**a**) and ECA109 (**b**) in a concentration‑ and time‑dependent manner (*P < 0.05, **P < 0.01, ns P > 0.05)

The proliferation rates of EC cell lines were determined using CCK‑8 assay. As shown in Fig. 1, CβC inhibited the proliferation of EC cells in a concentration‑ and time‑dependent manner. The 50% inhibitory concentration (IC50) values of CβC for TE-1 were 778.63 ± 52.79 μg/ml (24 h), 644.11 ± 12.37 μg/ml (48 h), and 410.64 ± 98.96 μg/ml (72 h). The IC50 values for ECA109 were 396.12 ± 32.07 μg/ml (24 h), 233.52 ± 10.83 μg/ml (48 h), and 130.51 ± 25.99 (72 h). Two low concentrations of CβC (10 μg/ml and 80 μg/ml) were chosen for the following experiments.

### CβC enhances radiosensitivity of EC cells under hypoxic and normoxic condition

**Fig. 2:**
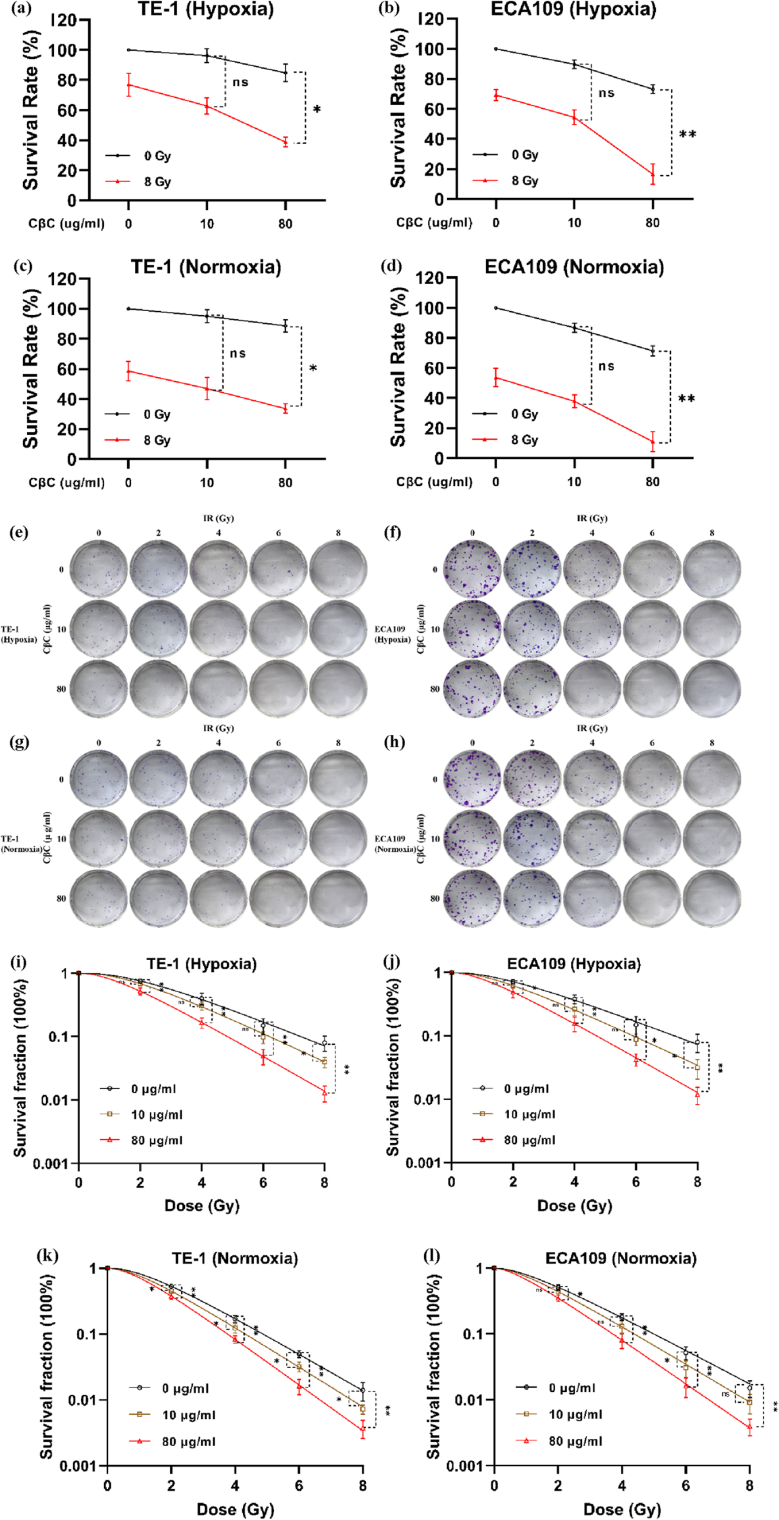
CβC enhanced radiosensitivity of EC cell lines under hypoxic and normoxic condition. (**a**–**d**) The CCK-8 assay showed that the viability of TE-1 and ECA109 cells decreased more markedly with the increase of CβC concentration after combined with irradiation (*P < 0.05, **P < 0.01, ns P > 0.05 versus the 0 Gy group). (**e**–**h**) The clonogenic survival assay showed that the number and size of cell colonies decreased significantly with the increases of irradiation dose and CβC concentration. (**i**–**l**) The survival curves were fitted according to the single-hit multi-target model. The cell survival fraction of the combined treatment group (CβC 80 μg/ml and irradiation) decreased more significantly than that of the irradiation alone group (*P < 0.05, **P < 0.01, ns P > 0.05 versus the 0 μg/ml group)

The radiosensitization effects of CβC in EC cell lines were determined using CCK‑8 assay and clonogenic survival assay. According to CCK-8 assay (Fig. 2a–d), the viability of EC cells decreased more markedly with the increase of CβC concentration after combined with irradiation, regardless of hypoxia or normoxia.

In the clonogenic survival assay (Fig. 2e–l), the cell survival fraction of the combined treatment group (CβC and irradiation) decreased more significantly than that of the irradiation alone group. According to the single-hit multi-target model, the D_0_, D_q_, SF_2_, and SERs were calculated and shown in Table 1. Together, these results reveal that CβC can enhance radiosensitivity under hypoxic and normoxic condition in EC cells.

**Table 1:** Radiosensitization effects of CβC in TE-1 and ECA109 cells

		CβC concentration	D_0_ (Gy)	D_q_ (Gy)	SF_2_ (%)	SER
Hypoxic	TE-1	0 μg/ml	2.17 ± 0.15	2.25 ± 0.40	0.75 ± 0.06	
		10 μg/ml	1.90 ± 0.10	1.89 ± 0.23	0.68 ± 0.04	1.14 ± 0.05^ ns^
		80 μg/ml	1.57 ± 0.06	1.26 ± 0.30	0.52 ± 0.07	1.39 ± 0.14^**^
	ECA109	0 μg/ml	2.32 ± 0.24	1.97 ± 0.28	0.71 ± 0.05	
		10 μg/ml	1.90 ± 0.16	1.57 ± 0.20	0.62 ± 0.05	1.22 ± 0.05^*^
		80 μg/ml	1.56 ± 0.08	1.13 ± 0.42	0.49 ± 0.09	1.48 ± 0.11^*^
Normoxic	TE-1	0 μg/ml	1.57 ± 0.23	1.30 ± 0.27	0.53 ± 0.03	
		10 μg/ml	1.43 ± 0.10	1.07 ± 0.12	0.45 ± 0.03	1.10 ± 0.14^ ns^
		80 μg/ml	1.25 ± 0.13	0.91 ± 0.29	0.38 ± 0.04	1.25 ± 0.06^*^
	ECA109	0 μg/ml	1.69 ± 0.01	1.38 ± 0.49	0.51 ± 0.05	
		10 μg/ml	1.49 ± 0.10	1.23 ± 0.59	0.44 ± 0.05	1.14 ± 0.08^ ns^
		80 μg/ml	1.29 ± 0.11	0.82 ± 0.15	0.35 ± 0.04	1.32 ± 0.11^**^

*CβC* Curcumol β-cyclodextrin inclusion complex, *D*_*0*_ Mean lethal dose, *D*_*q*_ Quasi-domain dose, *SF*_*2*_ Survival fraction at 2 Gy, *SER* Sensitization enhancement ratio. *P < 0.05, **P < 0.01, ns P > 0.05 versus the 0 μg/ml group

### CβC increases irradiation-induced apoptosis in EC cells

**Fig. 3:**
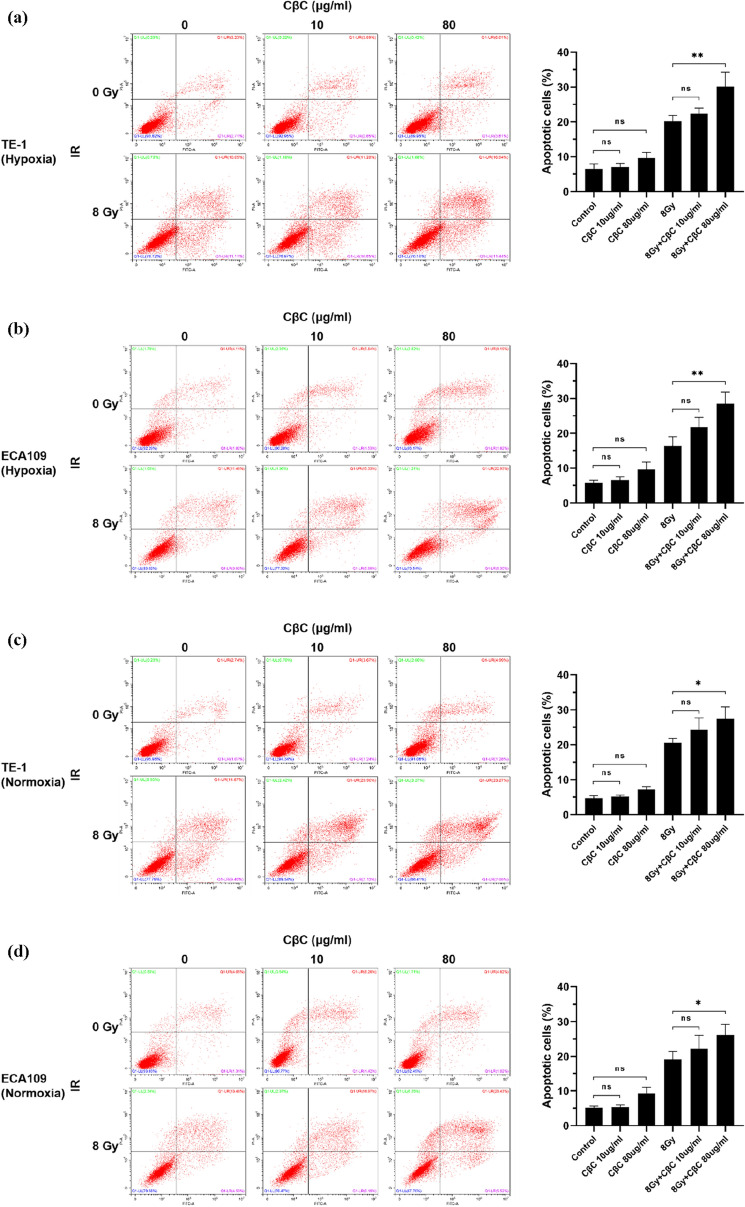

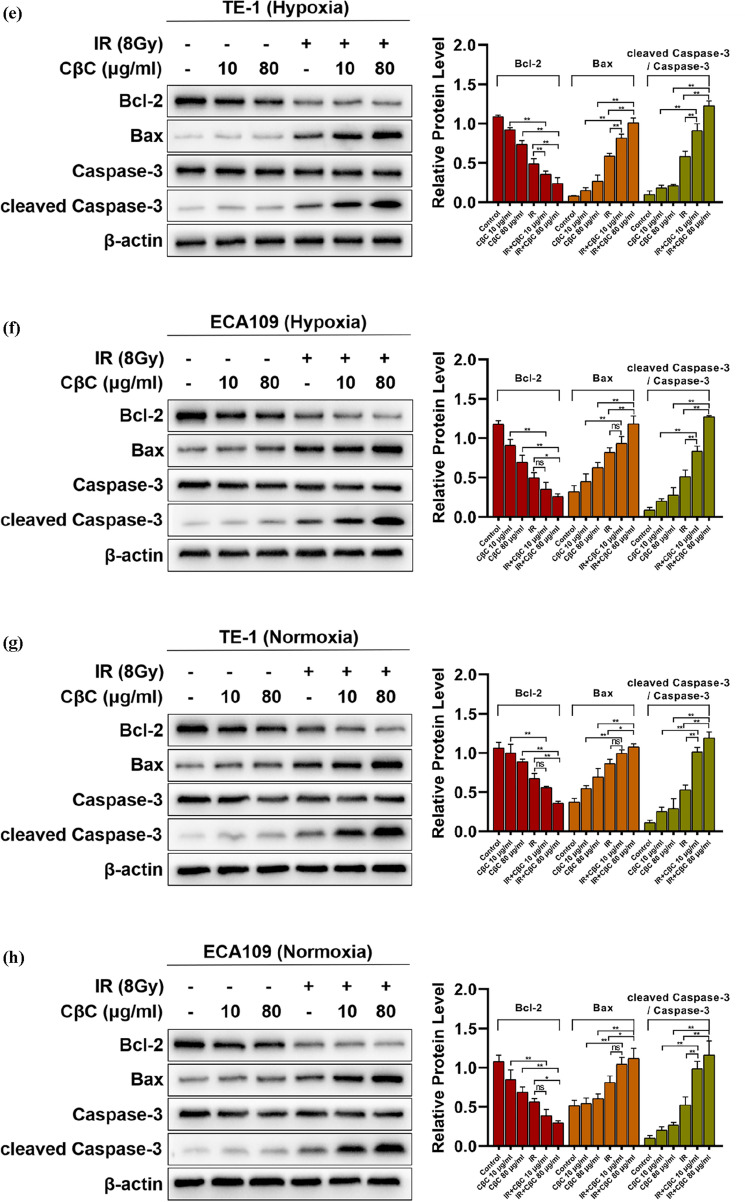
CβC increased irradiation-induced apoptosis in EC cell lines under hypoxic and normoxic condition. (**a**–**d**) The apoptosis ratio of the CβC group had no statistical significance compared with the control group (ns P > 0.05). The apoptosis ratio of irradiation combined with CβC 80 μg/ml was markedly higher than that of the irradiation group (*P < 0.05, **P < 0.01). (**e**–**h**) The combined treatment group (CβC 80 μg/ml and irradiation) significantly upregulated the expression of Bax and cleaved Caspase-3, and downregulated the expression of Bcl-2 (*P < 0.05, **P < 0.01, ns P > 0.05 versus the CβC group or irradiation group)

The proportion of apoptotic cells was detected by flow cytometer in EC cell lines. As shown in Fig. 3a–d, no matter in hypoxia or normoxia, the apoptosis ratio of the CβC group had no statistical significance compared with the control group, while the apoptosis ratio of irradiation combined with CβC 80 μg/ml was markedly higher than that of the irradiation group.

To further explore the molecular mechanisms, the expression of Bax, Bcl-2, Caspase-3, and cleaved Caspase-3 was examined. As shown in Fig. 3e–h, the expression of Bax, cleaved Caspase-3 was increased, whereas that of Bcl-2 was decreased in the combined treatment group (CβC and irradiation) compared with the single treatment group (CβC or irradiation). Collectively, the data reveal that CβC can increase irradiation-induced apoptosis under hypoxic and normoxic condition in EC cells.

### CβC increases the G2/M phase and inhibits DNA damage repair in EC cells

**Fig. 4:**
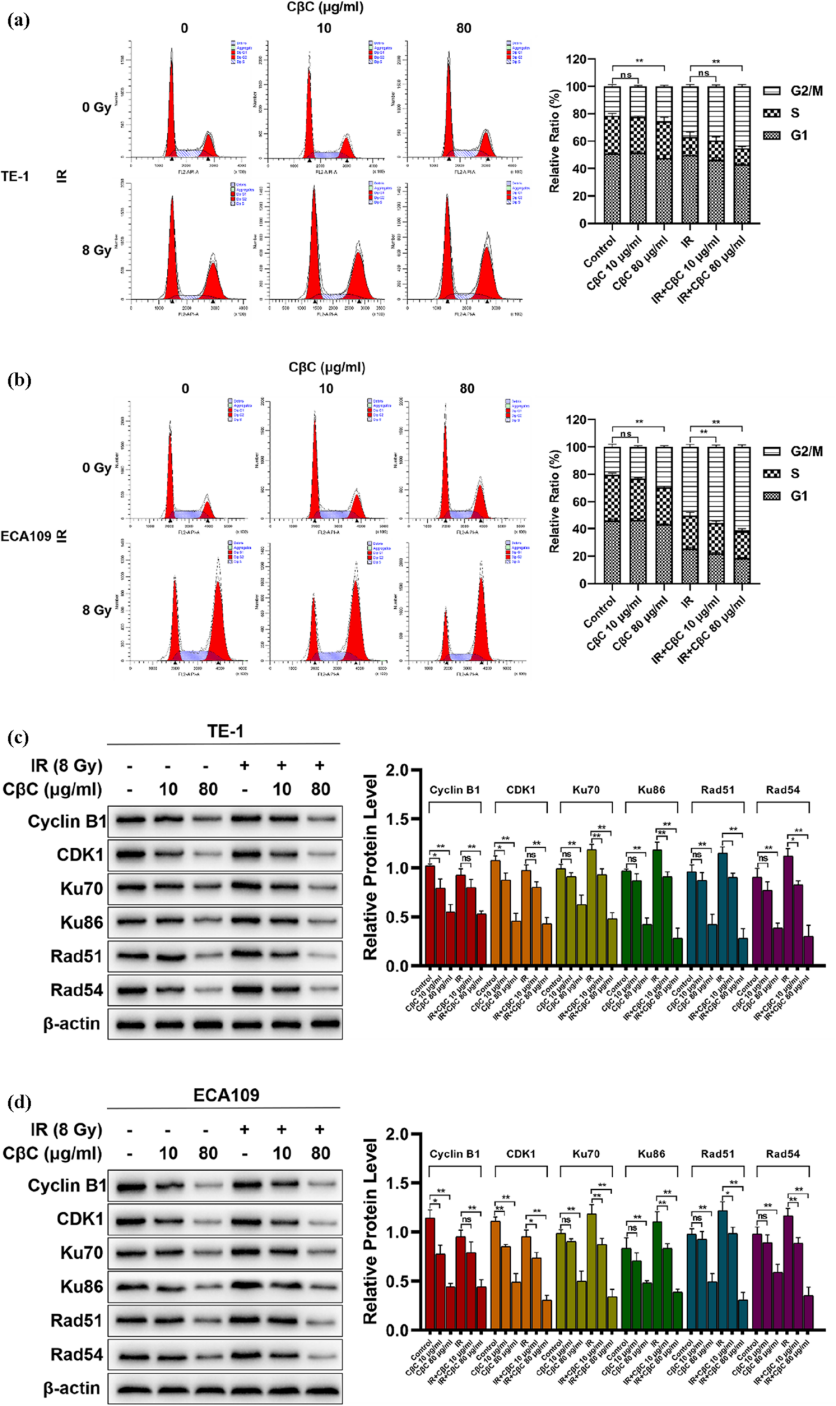
CβC increased the G2/M phase and inhibits DNA damage repair in EC cell lines. (**a**, **b**) CβC 80 μg/ml significantly blocked the cell cycle of EC cells in G2/M phase regardless of whether it was combined with irradiation (*P < 0.05, **P < 0.01, ns P > 0.05 versus the control group or irradiation group). (**c**, **d**) CβC 80 μg/ml significantly downregulated the expression of Cyclin B1 and CDK1 regardless of whether it was combined with irradiation (*P < 0.05, **P < 0.01, ns P > 0.05 versus the control group or irradiation group). CβC 80 μg/ml significantly downregulated the expression of Ku70, Ku86, Rad51 and Rad54 regardless of whether it was combined with irradiation (*P < 0.05, **P < 0.01, ns P > 0.05 versus the control group or irradiation group)

Flow cytometer was used to evaluate the cell cycle distribution of EC cell lines. As shown in Fig. 4a, b, CβC could markedly block the cell cycle of EC cells in G2/M phase regardless of whether it was combined with irradiation.

Cyclin B1 and CDK1 are important targets for cell cycle arrest in G2/M phase. As shown in Fig. 4c, d, the expression of Cyclin B1 and CDK1 was significantly decreased after CβC 80 μg/ml treatment, regardless of whether the EC cells were combined with irradiation. Ku70, Ku86, Rad51 and Rad54, as DNA damage repair-related proteins, were upregulated after irradiation, and down-regulated after CβC 80 μg/ml treatment. Thus, these results reveal that CβC can increase the G2/M phase and inhibit DNA damage repair in EC cell.

### Hypoxia-mediated upregulations of HIF‑1α and VEGF are attenuated by CβC through PI3K/Akt/mTOR signaling pathway

**Fig. 5:**
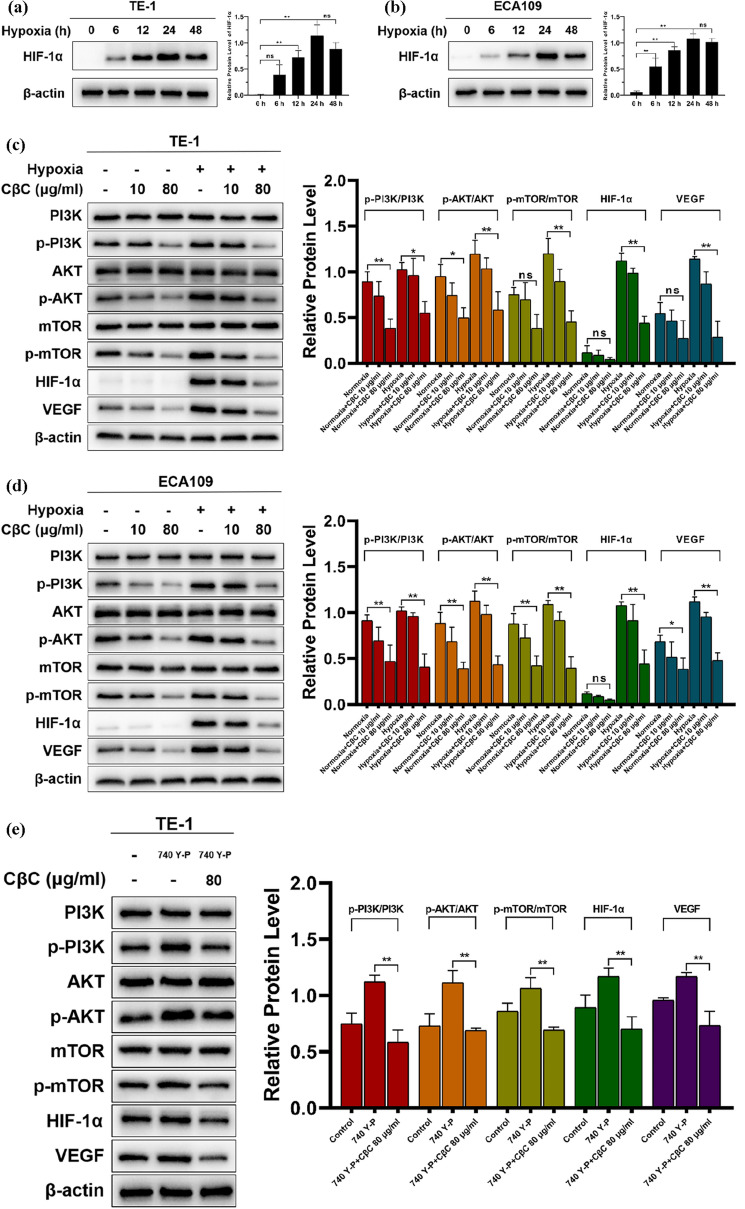

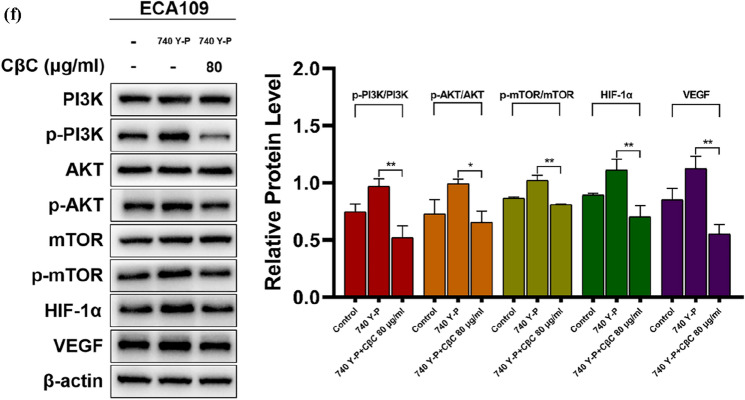
CβC attenuated hypoxia-mediated upregulations of HIF‑1α and VEGF through inhibiting the PI3K/Akt/mTOR signaling pathway in EC cell lines. (**a**, **b**) The protein expression of HIF‑1α was significantly upregulated at 6 h, and peaked at 24 h after hypoxic incubation (*P < 0.05, **P < 0.01, ns P > 0.05 versus the 0 h group or 48 h group). (**c**, **d**) The protein expression of p-PI3K, p-Akt, p-mTOR, HIF‑1α, and VEGF was upregulated under hypoxic condition, and CβC 80 μg/ml significantly downregulated the expression of these proteins (*P < 0.05, **P < 0.01, ns P > 0.05). (**e**, **f**) 740 Y-P upregulated the expression of p-PI3K, p-Akt, p-mTOR, HIF‑1α, and VEGF under hypoxia. CβC 80 μg/ml combined with 740 Y-P significantly downregulated these protein expression (*P < 0.05, **P < 0.01 versus the 740 Y-P group)

Figure 5a, b shows that the protein expression of HIF‑1α was significantly upregulated at 6 h, and peaked at 24 h after hypoxic incubation. Thus, hypoxia for 24 h was chosen for the following western blot assay. As shown in Fig. 5c, d the protein expression of p-PI3K, p-Akt, p-mTOR, HIF‑1α, and VEGF was upregulated under hypoxic condition, and CβC significantly attenuated the expression of these proteins.

The reverse verification assay was conducted under hypoxia and shown in Fig. 5e, f. 740 Y-P, as an activator of PI3K, further upregulated the expression of p-PI3K, p-Akt, p-mTOR, HIF‑1α, and VEGF under hypoxia. CβC could inhibit the effect of 740 Y-P and significantly downregulate the protein expression. These data indicate that CβC attenuates hypoxia-mediated upregulations of HIF‑1α and VEGF through inhibiting the PI3K/Akt/mTOR signaling pathway.

### CβC enhances radiosensitivity in vivo

**Fig. 6:**
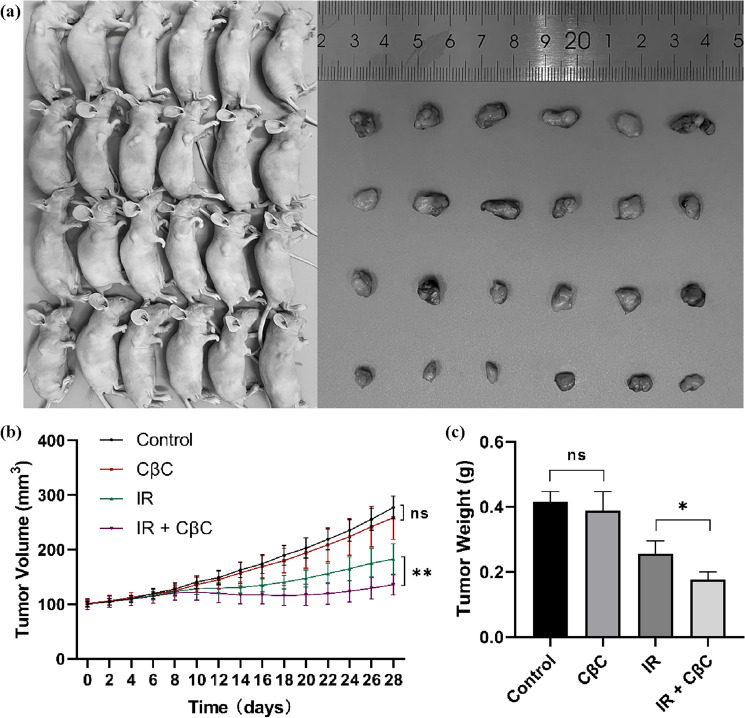
CβC enhanced irradiation-induced tumor growth inhibition in vivo. (**a**, **b**) The tumor volume of the CβC group had no statistical significance compared with the control group (ns P > 0.05). The tumor volume increased at a much slower rate in the combined treatment group (CβC and irradiation) compared with the irradiation group (**P < 0.01). (**c**) The tumor weight of the CβC group had no statistical significance compared with the control group (ns P > 0.05). The tumor weight of the combined treatment group (CβC and irradiation) was markedly lower than that of the irradiation group (*P < 0.05)

As shown in Fig. 6a, b, the tumor volume increased at a much slower rate in the combined treatment group (CβC and irradiation) compared with the irradiation group. As shown in Fig. 6c, the tumor weight of the combined treatment group (CβC and irradiation) was markedly lower than that of the irradiation group. The values of DT, AGD, NGD, and EF were shown in Table 2. The NGD of the combined treatment group was significantly higher than the AGD of the irradiation group, resulting in an EF of 2.45. These results reveal that CβC synergistically enhances irradiation-induced tumor growth inhibition in vivo.

**Table 2:** Radiosensitization effects of CβC in vivo

Group	DT	ADG	NGD	EF
Control	19.22 ± 1.23			
CβC	21.35 ± 3.02	2.13 ± 3.20		
IR	37.70 ± 16.36	18.48 ± 16.71		
IR + CβC	66.66 ± 11.38	47.43 ± 12.28	45.31 ± 13.12	2.45 (45.31/18.48)

*CβC* Curcumol β-cyclodextrin inclusion complex, *IR* Irradiation, *DT* Doubling time, *AGD* Absolute growth delay, *NGD* Normalized growth delay, *EF* Enhancement factor

## Discussion

Radiotherapy is widely used in the treatment of solid tumors, but the existence of radioresistant cells seriously affects its efficacy. In clinical practice, radioresistance is not uncommon in EC, and these patients often have a very poor prognosis. Therefore, to find an effective and safe radiosensitizer to enhance the radiosensitivity for EC is a matter of urgency for improving the efficacy of radiotherapy.

In recent years, more and more attention has turned to antitumor Chinese medicines [9]. Curcumol, as an active extract from traditional Chinese medicines, has been reported to have antitumor activity in various types of human tumor cells [4, 5], and can enhance radiosensitivity of Hela (human cervical carcinoma cells), K-562 (human chronic myelogenous leukemia cells), and IM-9 (human peripheral blood B lymphocytes) cell lines [10]. However, its reversal of hypoxia-related radioresistance has been rarely reported. In the present study, curcumol was prepared as an inclusion complex with β-cyclodextrin by saturated solution method, and its antitumor activity was retained. In vivo experiments by Caillaud et al., the average plasma concentration of curmumol was higher after intraperitoneal injection of CβC than that of free curmumol. After intraperitoneal injection of CβC, the highest plasma concentration appeared at 1 h and could not be measured at 8 h [11]. This is the first study to demonstrate the radiosensitization effect of CβC in EC cells under hypoxic and normoxic condition. The effect of radiosensitization of CβC was investigated in vitro and in vivo in this paper. The in vitro data revealed that CβC and irradiation synergistically inhibited the proliferation, reduced the colony formation, promoted the apoptosis, increased the G2/M phase, inhibited DNA damage repair, and reversed the hypoxia-mediated radioresistance of EC cells to a greater extent than did CβC alone or irradiation alone. The SERs were 1.39 for TE-1 and 1.48 for ECA109 under hypoxia. The SERs were 1.25 for TE-1 and 1.32 for ECA109 under normoxia. The in vivo data demonstrated that the combination of CβC and irradiation could inhibit tumor growth to the greatest extent compared with either monotherapy alone. The EF was 2.45. Taken together, the above data strongly indicate that CβC has a radiosensitization effect on EC cells under hypoxia and normoxia.

Apoptosis, as a major process of cell death following irradiation, is activated via the extrinsic pathway (death receptor pathway) or the intrinsic pathway (mitochondrial death pathway) [12, 13]. The Bcl-2 family includes anti-apoptotic protein Bcl-2 and pro-apoptotic protein Bax, and plays a central role in intrinsic pathway [14]. Bcl-2 and Bax not only mediate radiotherapy-induced cell death, but also regulate cancer radiosensitivity. The increased ratio of Bax/Bcl-2 may enhance sensitivity of cancer radiotherapy [15–18]. Caspase-3 is the main executor of both intrinsic and extrinsic pathways and is a frequently activated death protease, catalyzing the specific cleavage of many key cellular proteins [19]. In this study, the apoptosis ratio of irradiation combined with CβC 80 μg/ml was markedly higher than that of the irradiation group in hypoxia and normoxia. Subsequently, the western blot assay showed that the expression of Bax and cleaved caspase-3 was significantly increased, while the expression of Bcl-2 was significantly decreased in the combined treatment group compared with the monotherapy groups. Accordingly, the results indicate that CβC combined with irradiation can induce more apoptosis of EC cells through the intrinsic apoptosis pathway.

The cell cycle is divided into G1 phase (Gap period I), S phase (DNA synthesis), G2 phase (Gap period II), and M phase (Mitosis). Tumor cells in different cell cycle phases have different radiosensitivity. Cells in G2 and M phases are the most sensitive to radiotherapy, while cells in S phase are the least sensitive to radiotherapy [20, 21]. Increasing the ratio of tumor cells in G2/M phase can effectively sensitize radiotherapy [22–24]. The cell cycle is regulated by CDKs and cyclins, of which the Cyclin B1-CDK1 complex is critical for regulating cell entry into mitosis [25]. In this study, CβC significantly increased the G2/M phase ratio of EC cells. Subsequently, the western blot assay showed that the expression of Cyclin B1 and CDK1 was significantly decreased in CβC treatment group. Collectively, the results indicate that CβC can arrest the cell cycle in G2/M phase by inhibiting the expression of Cyclin B1 and CDK1 in EC cells.

DNA double-strand breaks play a major role in the radiation-induced killing of tumor cells. DNA double strand damage repair, including non-homologous end joining (NHEJ) and homologous recombination (HR), severely affects radiosensitivity. Ku70 and Ku86 are key proteins of NHEJ, and RAD51 and RAD54 are key proteins of HR. When DNA double strand breaks, these proteins gather around the broken ends to repair DNA. Thus, overexpression of Ku70, Ku86, RAD51 and RAD54 can lead to enhanced DNA damage repair and radioresistance. It has been documented that downregulation of these proteins can sensitize radiotherapy in multiple cancer types [26–29]. In this study, Ku70, Ku86, Rad51 and Rad54 were up-regulated after irradiation, and down-regulated after CβC 80 μg/ml treatment. Thus, these results reveal that CβC can inhibit DNA damage repair by simultaneously attenuating NHEJ and HR in EC cells.

Hypoxia state is common in solid tumors and plays a crucial role in radioresistance. To adapt to the hypoxic microenvironment, tumor cells modulate the signaling pathways that influence development, metabolism, inflammation, and integrative physiology, thus further promoting angiogenesis, invasion, metastasis, and resistance to radiatiotherapy and chemotherapy [30, 31]. Radiotherapy is an indispensable treatment for EC, but its efficacy is affected by hypoxia state [32].

As a heterodimeric transcription factor, hypoxia-inducible factor 1 (HIF-1) is composed of two different subunits: an oxygen-sensitive α-subunit (HIF-1α) and a constitutively present β-subunit (HIF-1β). Under hypoxic condition, HIF-1α cannot be effectively degraded and substantially accumulates in tumor cells, and then HIF-1α binds to HIF-1β, forming a functional heterodimer, HIF-1 [30, 33]. HIF-1 regulates the expression of numerous genes through binding to DNA at hypoxia response elements and helps tumor cells adapt to hypoxic microenvironment, which promotes tumor progression, metastasis, and resistance to radiotherapy [34–38]. HIF-1α, as the marker protein of hypoxia, is not only influenced by hypoxic condition, but also regulated by PI3K/Akt/mTOR signaling pathway [39–41]. The PI3K/Akt/mTOR signaling pathway regulates the synthesis and stabilization of HIF-1α in hypoxic tumor cells [41]. HIF‑1α promotes the expression of VEGF, which leads to tumor angiogenesis and increases metastatic potential [42, 43]. HIF-1α and VEGF have great clinical significance for the prediction of radiochemotherapy response and prognosis [44].

PI3K is activated by receptor tyrosine kinases (RTKs) or G-protein coupled receptors (GPCRs). After that the activated PI3K phosphorylates the phosphatidylinositol 4,5-phosphate (PIP2) into phosphatidylinositol 3,4,5-trisphosphate (PIP3). PIP3, as a second messenger, binds to the pleckstrin homology (PH) domain of Akt, and then Akt is phosphorylated at Thr308 by phosphoinositide-dependent kinase-1 (PDK1) and Ser473 by mammalian target of rapamycin complex 2 (mTORC2). Subsequently, as a GTPase activating protein, tuberous sclerosis complex 2 (TSC2) is inhibited by activated Akt, and then Rheb‐GTP activates mTORC1 [45–47]. Finally, the mTORC1-mediated signaling pathway stimulates the synthesis of HIF-1α in hypoxic tumor cells [33, 34, 41, 47]. Several papers have reported that PI3K/Akt/mTOR is activated under hypoxic condition, which further upregulates HIF-1α expression [48–50]. Herein, we verified again that the expression of p-PI3K, p-Akt, p-mTOR, HIF‑1α, and VEGF was upregulated under hypoxic condition, and demonstrated that CβC could attenuate the expression of these proteins under hypoxic condition. According to the reverse validation assay, the data demonstrate that CβC can downregulate the expression levels of HIF‑1α and VEGF through targeting the PI3K/Akt/mTOR signaling pathway.

## Conclusions

**Fig. 7:**
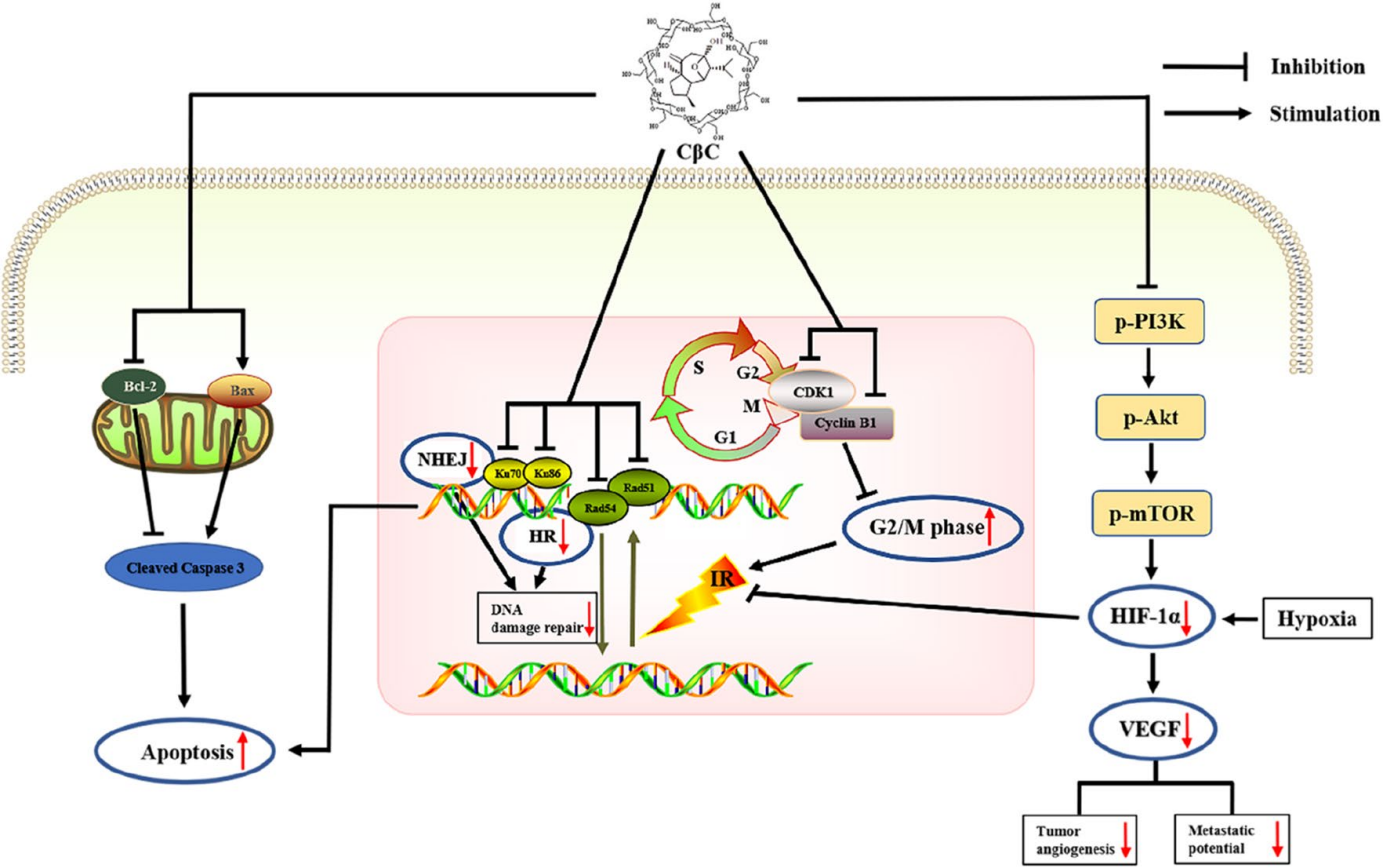
CβC enhances radiosensitivity of EC cells under hypoxic and normoxic condition through increasing the irradiation-induced apoptosis, arresting the cell cycle in G2/M phase, inhibiting DNA damage repair, and downregulating HIF‑1α and VEGF expression

In summary, these results well complement the previous related researches, and promote further understanding of the mechanism of CβC. This study demonstrated that CβC could enhance radiosensitivity of EC cells under hypoxic and normoxic condition through increasing the irradiation-induced apoptosis, arresting the cell cycle in G2/M phase, inhibiting DNA damage repair, and downregulating HIF‑1α and VEGF expression (Fig. 7). Thus, CβC can be used as an effective radiosensitizer for EC. Future studies are warranted to explore the deeper mechanisms of radiosensitization effect of CβC.
